# Editorial: Consequences and aftercare of a traumatic loss of a loved one

**DOI:** 10.3389/fpsyt.2022.1111000

**Published:** 2023-01-05

**Authors:** Lonneke I. M. Lenferink, Josefin Sveen, Fiona Maccallum

**Affiliations:** ^1^Department of Psychology, Health and Technology, Faculty of Behavioural Management and Social Sciences, University of Twente, Enschede, Netherlands; ^2^Department of Clinical Psychology, Faculty of Social and Behavioural Sciences, Utrecht University, Utrecht, Netherlands; ^3^Department of Clinical Psychology and Experimental Psychopathology, Faculty of Behavioral and Social Sciences, University of Groningen, Groningen, Netherlands; ^4^Centre for Crisis Psychology, Faculty of Psychology, University of Bergen, Bergen, Norway; ^5^Department of Women's and Children's Health, Uppsala University, Uppsala University Hospital, Uppsala, Sweden; ^6^School of Psychology, The University of Queensland, St. Lucia, QLD, Australia

**Keywords:** grief, bereavement, loss, death, trauma

The potential long-term negative health impacts associated with bereavement have been recognized with the entry of Prolonged Grief Disorder (PGD) into the most recent editions of the Diagnostic and Statistical Manual of Mental Disorders (DSM-5-TR) (1) and International Classification of Diseases (ICD-11) (2). People who are confronted with the traumatic loss of a loved one, such as a sudden, violent, or unnatural loss, are at greater risk of developing mental disorders, including PGD, posttraumatic stress disorder (PTSD), and depression than non-traumatically bereaved people (3–6). In this special issue, we are pleased to present nine articles on the consequences and aftercare of a (traumatic) loss of a loved one. Comprising quantitative, qualitative, and theoretical approaches, the papers were (co-)written by 42 authors from institutes based in Europe, North America, and Oceania; Eight of nine leading authors were women. Included in the empirical studies are 7,763 bereaved adolescents/adults who have experienced varied losses including a death during the COVID-19 pandemic (Dominguez-Rodriguez et al.), death of a child (Eklund et al.), and the long-term disappearance of a family member (Wayland and Ward). The papers also challenge the field to think about the extent to which currently accepted findings apply across individuals with different cultural backgrounds including Arab and Sub-Saharan Africans with a refugee background (Lechner-Meichsner and Comtesse) and black Americans (Wilson and O'Connor).

Four studies focus on severity of distress post-loss. In a Mexican sample (*N* = 5,224) prevalence rates and correlates of survey-based distress were examined in people who accessed an online support platform for bereavement during the pandemic. Two thirds of participants reported problematic grief reactions and 90% reported clinically relevant anxiety, depression, and/or sleep problems. A recent suicide attempt was the strongest correlate of post-loss distress (Dominguez-Rodriguez et al.). This study suggests that bereavement during the pandemic may increase the risk for post-loss distress.

In a study including a convenience sample of 433 Dutch and German bereaved adults interviewed by telephone, Heeke et al. examined patterns of comorbidity among PGD, PTSD, and depression symptoms using latent class analysis. This analysis identified three classes that differed in symptom intensity, rather than being qualitatively different. Characteristics often observed in traumatic loss, including unexpectedness of the loss and less meaning making were associated with classes with more pervasive distress levels.

Doering et al. further investigated the role of unexpectedness in a representative German sample (*N* = 811). Loss of a child was most strongly related to PGD outcomes, however, perceived unexpectedness of the death was associated with both PGD caseness and PGD severity. Together, these studies highlight features of traumatic loss that can place individuals at risk for poor outcomes.

Additionally, Mitima-Verloop et al. investigated a different set of risk factors for poor bereavement outcomes, namely the inability to undertake culturally accepted grief rituals in a diverse bereaved sample (representing 32 nationalities mostly from European countries). In their timely cross-country survey-study they compared disturbed grief levels and evaluations of funeral rituals between people who experienced a loss before (*N* = 50) or during the pandemic (*N* = 182). While a more negative general funeral evaluation was related to higher grief levels, grief levels and funeral evaluations did not differ between the two groups (Mitima-Verloop et al.). Their findings highlight the overall importance of funeral practices and cultural rituals around death.

Two papers investigate processes that may be targeted to reduce grief related distress. Eklund et al. pilot-tested a self-help mobile-app (based on cognitive-behavioral theories) providing parents who had lost a child with psychoeducation, support, and exercises such as exposure to avoided aspects of the loss (*N* = 13), using a mixed-method design. The app seemed feasible and acceptable to participants and preliminary findings showed decreases in symptoms across the trial. While this paper highlights the need for interventions targeting (prolonged) grief, especially early in the grief process, and how technology can be applied to improving grief outcomes, more research in larger samples is needed.

Another important cognitive process underlying PGD is poor loss-related memory integration. Smith et al. described the development and evaluation of a scale to measure loss-related memory characteristics in three independent bereaved community samples (total *N* = 1,001). The scale showed excellent psychometric properties, and a cross-lagged panel analysis showed that memory characteristics predicted later PGD symptoms. The scale offers a new tool for targeting memories during PGD treatment and should facilitate novel research on memory processes in grief.

Two studies consider whether current conceptualization of grief can be applied cross-culturally. Lechner-Meichsner and Comtesse used a mixed-methods approach to explore beliefs about PGD among Arab and Sub-Saharan African refugees living in Germany. Similarities with western conceptualizations regarding causes and “cures” were found, such as suddenness of the death and needs for emotional processing. However, potential cultural and context-specific features relevant to help-seeking behaviors were also identified. Findings highlight the importance of a culture-sensitive treatment-approach for PGD in refugees. Wilson and O'Connor question the application of current conceptualizations of grief, which focus only on the individual, across cultures. In this thought-provoking piece, they outline a novel theoretical model for understanding collective grief amongst Black Americans, a group regularly underrepresented in the literature. The model describes how the historical and ongoing racial violence, economic dispossession and structural inequality experienced by black Americans has shaped a grief that is both quantitatively and qualitatively different from current conceptualizations as of grief as an individual response to loss. In recognizing the collective nature of grief, the model also proposes an important role for grief as a catalyst for social action for black Americans.

Wayland and Ward expand the discussion of grief further to explore the experiences of Australian families living with the ambiguous loss of a missing person. This mixed-methods paper focused on the experience of providing a DNA sample for the purpose of body identification. The authors draw attention to a range of contextual and procedural factors that can exacerbate or lessen the distress associated with this process, suggesting important procedural changes to improve the system for relatives of missing people.

Grief is a complex phenomenon. The inclusion of a formal PGD diagnosis in classification systems provides an important opportunity to consider how we, as a field, would like to progress. Taken together, we anticipate this diverse set of papers will enhance knowledge on assessment, prediction, and treatment of distress in traumatically bereaved people, as well as highlight similarities and differences across cultures and contexts. We are pleased to see the increased application of sophisticated statistical techniques and mobile technology to study grief *in situ* and provide support to individuals in their daily lives. We hope that international collaborations, such as this special issue, and the ongoing application of rigorous and varied methodological approaches will continue to offer significant potential to improve outcomes for those struggling following the loss of a loved one.

## Author contributions

LL, JS, and FM co-edited this research topic. LL wrote the first draft of the manuscript. JS and FM wrote sections of the manuscript. All authors contributed to manuscript revision, read, and approved the submitted version.
